# DRQuantum: a drug repurposing method by quantum walks on a multi-layered heterogeneous network

**DOI:** 10.1038/s41598-026-58022-y

**Published:** 2026-06-15

**Authors:** Zengjing Chen, Xin Guo, Hao Jiang, Zhiping Liu, Ziqi Lu

**Affiliations:** 1 Shandong National Center for Applied Mathematics, Shandong University, Jinan, 250100 China; 2 Department of Mathematics, Shandong University, Jinan, 250100 China; 3 Zhongtai Securities Institute for Financial Studies, Shandong University, Jinan, 250100 China

**Keywords:** Drug repurposing, Quantum walks, Network embedding, Heterogeneous networks, Computational biology and bioinformatics, Drug discovery, Mathematics and computing, Physics

## Abstract

Traditional drug development poses significant financial and temporal costs, whereas drug repurposing emerges as a cost-effective and efficient alternative. As large-scale biological networks proliferate, computational drug repurposing has become feasible, yet accurately capturing intricate heterogeneous network structures remains a persistent challenge. To address this challenge, we introduced a novel approach, called DRQuantum: Drug Repurposing via Quantum walks. Unlike random walks, quantum walks dispense with independence and harness quantum entanglement to simultaneously explore multiple paths, enabling faster traversal of networks. Moreover, DRQuantum accounts for both the local and global network structures. In this study, we constructed a heterogeneous multi-layer network by integrating drug-drug, disease-disease and protein-protein interaction networks. We then employed quantum walks to learn low-dimensional feature representations of nodes in these heterogeneous networks, ultimately inferring candidate drugs for repurposing beyond their original indications. Consequently, we observed that DRQuantum outperforms traditional drug repurposing methods in terms of AUROC, AUPRC and accuracy. Additionally, case studies for several specific diseases further validate the practical utility of our proposed method.

## Introduction

Drug repurposing has emerged as a pivotal strategy in drug development^1,2^, particularly notable in addressing urgent health crises like COVID-19^3,4^. Traditional drug development procedures are often lengthy and expensive, involving extensive years and substantial financial resources^5,6^. Conversely, drug repurposing capitalizes on existing drugs with proven safety profiles to discover novel therapeutic applications, offering a more efficient approach. Numerous drugs originally intended for treating diverse diseases have been successfully repurposed and approved for new indications^1,2,7,8^. This strategy not only expedites the accessibility of treatments but also substantially mitigates the costs and risks associated with clinical trials.

With the swift advancement of large-scale biological networks and diverse biological databases, computational pharmacology has emerged as a powerful tool for drug repurposing studies. Currently, numerous sophisticated models are being utilized to investigate novel therapeutic avenues for drugs. The available methods proposed for drug repurposing can be broadly grouped into four categories: similarity- and matrix-based methods^9,10^, random walk-based methods^11–13^, knowledge graph embedding-based methods^14,15^, and deep learning and graph neural network (GNN)-based methods^16–18^. More recently, advanced GNN architectures incorporating heterogeneous graph learning and attention mechanisms have further pushed the boundary of drug–disease association prediction^19–21^, and large language model (LLM)-enhanced approaches have begun to emerge as a promising new direction^22^. However, these models encounter significant challenges in practical applications. Firstly, a pivotal challenge lies in learning informative, low-dimensional representations of networks while preserving the heterogeneity of drug-related networks. In these biological networks, drugs with similar or identical functional annotations often exhibit intricate relationships that originate from homophily and structural similarity. Therefore, devising algorithms capable of accurately capturing these intricate network structures while maintaining predictive prowess for novel drug indications is highly demanding. Additionally, biological networks constitute vast, highly nonlinear heterogeneous networks encompassing thousands of nodes. In such intricate environments, rapidly and comprehensively learning network structures while optimizing storage space and computational resources utilization remains a prominent limitation of current algorithms.

Discrete-time quantum walks (DTQW), originally proposed by Aharonov^23^, represent a potentially powerful technique for modelling the intricacies of extensive, highly non-linear heterogeneous networks. Serving as a cornerstone model in quantum computing, quantum walks constitute the quantum analog of classical random walks, thereby attracting attention as a viable alternative approach. Distinct from classical random walks, quantum walks do not assume independence; the walker exists in a superposed state characterized by quantum entanglement, simultaneously traversing all conceivable paths. This attribute ensures the retention of path information and the efficient capture local structures by quantum walks. Numerous quantum algorithms, including Grover’s search algorithm^24^ and graph traversal algorithms^25^, have emerged from quantum walk principles. When benchmarked against the most advanced classic algorithms addressing the same challenges, these quantum algorithms exhibit varying degrees of acceleration. Konno’s research^26^ revealed that on a one-dimensional line, a quantum walk propagates at a speed of *O*(*N*). Typically, searching for an entry in an unsorted database demands *O*(*N*) time to sift through *N* items. However, by integrating the quantum walk mechanism, Grover’s algorithm enhances the search time to $O(\sqrt{N})$. Additionally, in the context of graph-based quantum walks, a quadratic acceleration effect is evident in hitting times^27^, a phenomenon that extends to quantum walks on diverse structures^28–30^. Furthermore, due to its high-density information encoding capability, quantum computing theoretically necessitates less storage space for processing extensive datasets^31,32^.

In this paper, we present a unique method that employs quantum walk algorithms to efficiently embed drug and disease nodes within multi-layer networks. The objective is to capture intricate relationships between nodes, thereby enhancing the precision of drug repurposing predictions. To ensure effective identification of potential drug-disease associations after embedding enhancement, we utilize clustering analysis and the XGBoost classifier^33^. This study introduces a sophisticated drug repurposing analysis framework via quantum walks on a heterogeneous multilayer biomolecular network aimed at boosting prediction accuracy and efficiency.

## Results

### Overview the methods of DRQuantum

**Fig. 1:**
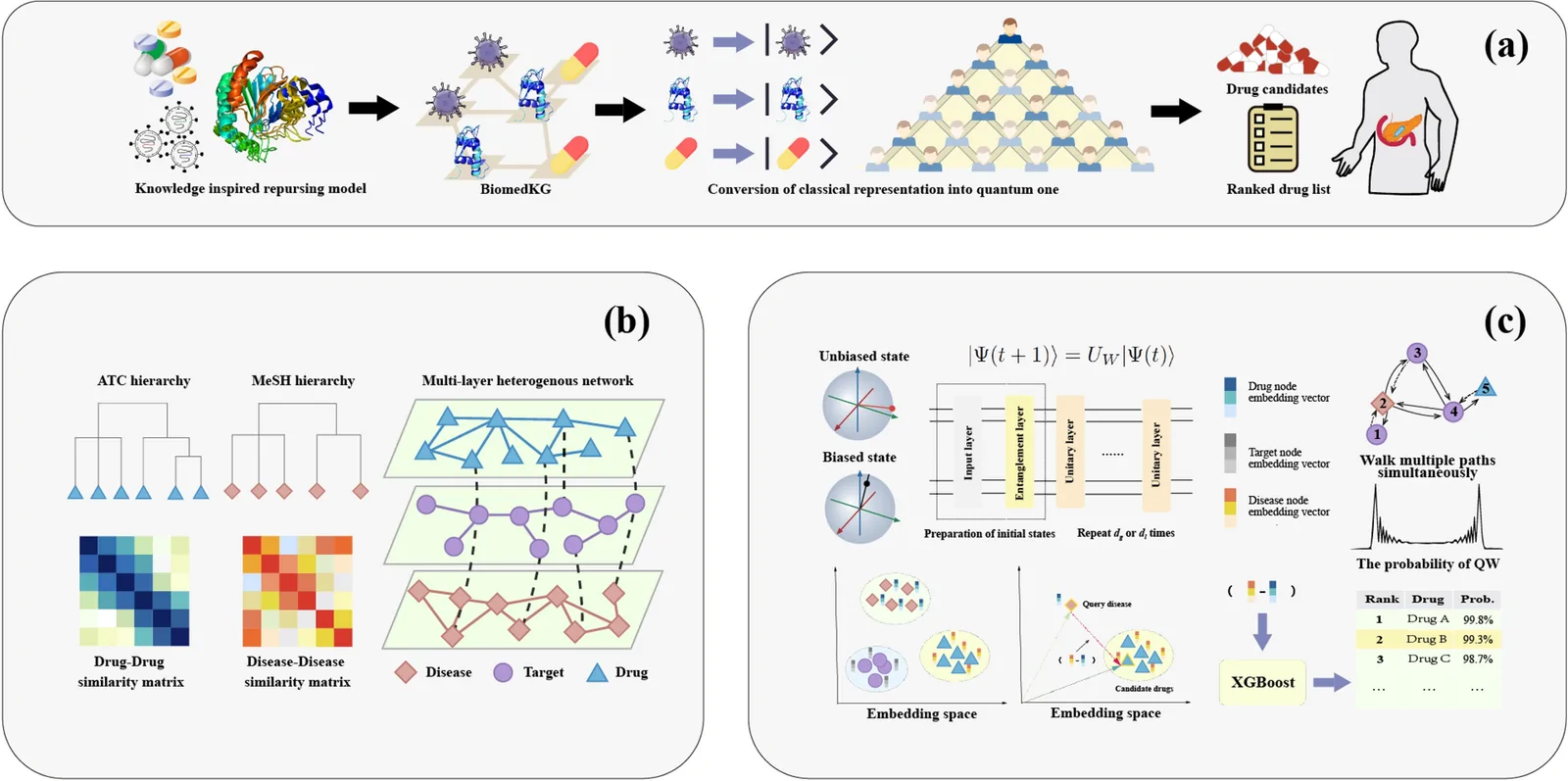
(**a**) The framework of DRQuantum. (**b**) Calculating drug-drug and disease-disease similarity matrices utilizing the ATC and MeSH hierarchical classifications respectively, with the objective of constructing a multi-layer heterogeneous network encompassing drugs, targets and diseases. (**c**) Performing quantum walks on the constructed drug-target-disease network to generate quantum state sequences. These quantum states sequences are then encoded to produce node embedding vectors. An XGBoost classifier is employed, taking the difference between the drug and disease embedding vectors as input, to predict the probability of corresponding treatment efficacy.

In biological networks, adjacent biological entities often exhibit similar biological traits, a concept referred to as “guilt by association”^34^. This idea implies that in biomolecular networks, such as protein-protein interaction networks or gene regulatory networks, directly connected biological entities (e.g., proteins or genes) tend to have comparable functions. In the realm of drug repurposing research, associations between drugs and diseases are frequently explored through biological targets serving as bridges. This involves networks at multiple levels, including drug and disease networks. Hence, we introduce the DRQuantum algorithm, which incorporates the “guilt by association” principle within multi-layer heterogeneous networks, specifically drug-target-disease networks. By employing quantum walks, the DRQuantum algorithm efficiently and flexibly captures both the local node structure and the overall network structure, enabling the inference of drug-disease relationships. Fig. 1 provides an overview of the DRQuantum algorithm’s framework, and detailed explanations of the technologies utilized in the DRQuantum algorithm can be found in Methods section.

*Step 1* **Similarity matrices and initial quantum walk state:** We initiate the process by computing similarity matrices for drugs and diseases, using the commonly accepted Anatomical Therapeutic Chemical (ATC) classification for drugs and Medical Subject Headings (MeSH) for diseases. These similarity matrices are then used to construct a multi-layer network connecting drugs, targets, and diseases based on their respective node similarities.

*Step 2* **Quantum walk-based node embedding (RED algorithm**^35^): The quantum walk generates a sequence of quantum states that encapsulates the network’s structural information. By encoding the amplitudes of these states, we obtain an embedding representation for each node. These embeddings integrate both local and global structural features of the nodes. Within the embedding space, we can measure the distance between nodes, allowing us to quantify and compare similarities or differences between various entities, thereby facilitating downstream tasks.

*Step 3* **Predicting drug-disease treatment probability with XGBoost:** Using the generated node representations, we train an XGBoost classifier to predict the treatment probability of a drug-disease pair. The prediction is based on the difference vector between their respective node embeddings. The trained XGBoost model aids in drug repurposing by highlighting drug-disease relationships with high treatment probabilities. Detailed information about the model can be found in Methods.

### Effectiveness

In this section, we evaluated DRQuantum against five representative baseline methods on both the MSI and HetioNet datasets. The selected baseline methods included BNNR^9^, PREDICT^10^, DeepDR^16^, RGCN^17^, and NIMCGCN^18^, which represent several mainstream paradigms for drug–disease association (DDA) prediction.

Among these methods, BNNR and PREDICT belong to traditional matrix- and similarity-based approaches. Specifically, PREDICT infers potential drug–disease associations by exploiting drug similarity and disease similarity information, whereas BNNR employs bounded nuclear norm regularization and matrix completion techniques to recover missing associations from incomplete interaction matrices. In contrast, DeepDR, RGCN, and NIMCGCN are deep learning-based approaches designed for heterogeneous biomedical networks. DeepDR utilizes deep neural architectures to integrate multiple biological data sources and learn latent representations for drug repositioning tasks. RGCN adopts relational graph convolutional networks to explicitly model multi-relational dependencies among biomedical entities. NIMCGCN further incorporates neighborhood interaction mechanisms into graph convolutional networks to simultaneously capture local topological structures and global association patterns within biological networks. To ensure a fair comparison, all competing methods were implemented under identical experimental settings and evaluated using standard metrics, including AUROC, AUPRC, and Accuracy. All known drug–disease associations used for evaluation were strictly excluded from the training process during cross-validation to avoid information leakage.

**Fig. 2:**
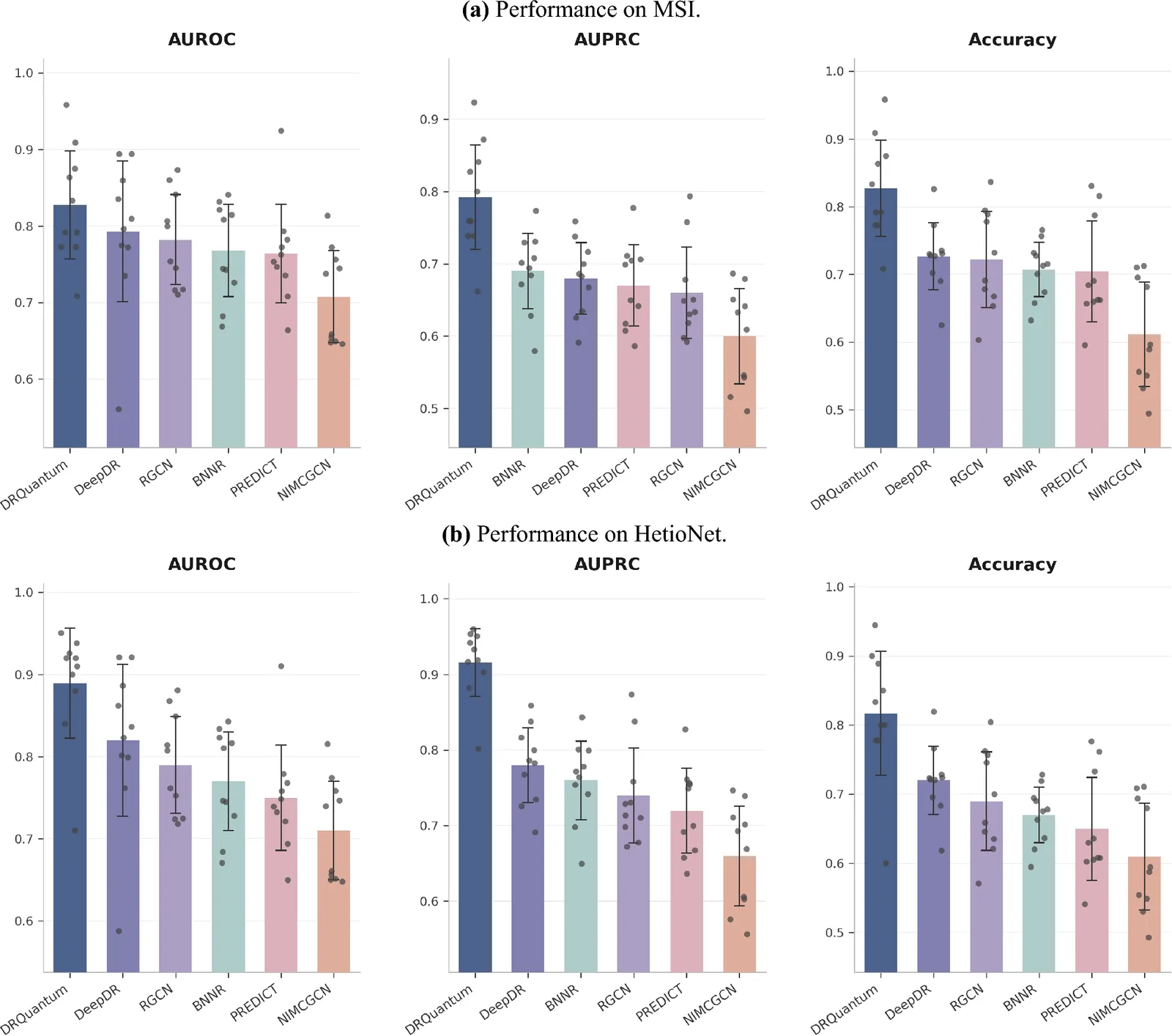
The DDA performances of each model on the two biomedKGs. Bar height represents the mean value derived through *n=10* independent experiments, and error bars denote the standard deviation across 10-fold cross-validation experiments. Individual dots represent results from each repetition.

As shown in Fig. 2, DRQuantum consistently achieves the best overall performance across two different datasets and evaluation criteria, with an AUROC of 0.828, AUPRC of 0.792, and Accuracy of 0.828 on MSI, and an AUROC of 0.890, AUPRC of 0.916, and Accuracy of 0.817 on HetioNet.

**Vs. traditional similarity-based methods:** PREDICT and BNNR perform comparably to each other on both networks, with PREDICT achieving an AUROC of 0.764 on MSI and 0.750 on HetioNet, and BNNR achieving an AUROC of 0.768 on MSI and 0.770 on HetioNet. Their AUPRC values are similarly limited, reaching 0.670 and 0.690 on MSI, and 0.720 and 0.760 on HetioNet, respectively. The gap relative to DRQuantum is largest in AUPRC, up to 0.22 on MSI, confirming that hand-crafted similarity measures are insufficient for recovering sparse positive associations in heterogeneous biological networks.

**Vs. deep learning-based methods:** Among deep learning baselines, DeepDR achieves the strongest AUROC of 0.793 on MSI and 0.820 on HetioNet, while RGCN and NIMCGCN trail behind with an AUROC of 0.782 and 0.708 on MSI, and 0.790 and 0.710 on HetioNet. In AUPRC, however, all three methods fall substantially below DRQuantum, with DeepDR reaching an AUPRC of only 0.680 on MSI and 0.780 on HetioNet. DRQuantum consistently surpasses this group by margins of 0.03–0.19 in AUPRC across both datasets, suggesting that quantum-walk-based representations capture drug–disease associations more discriminatively than standard graph neural network architectures.

**Fig. 3:**
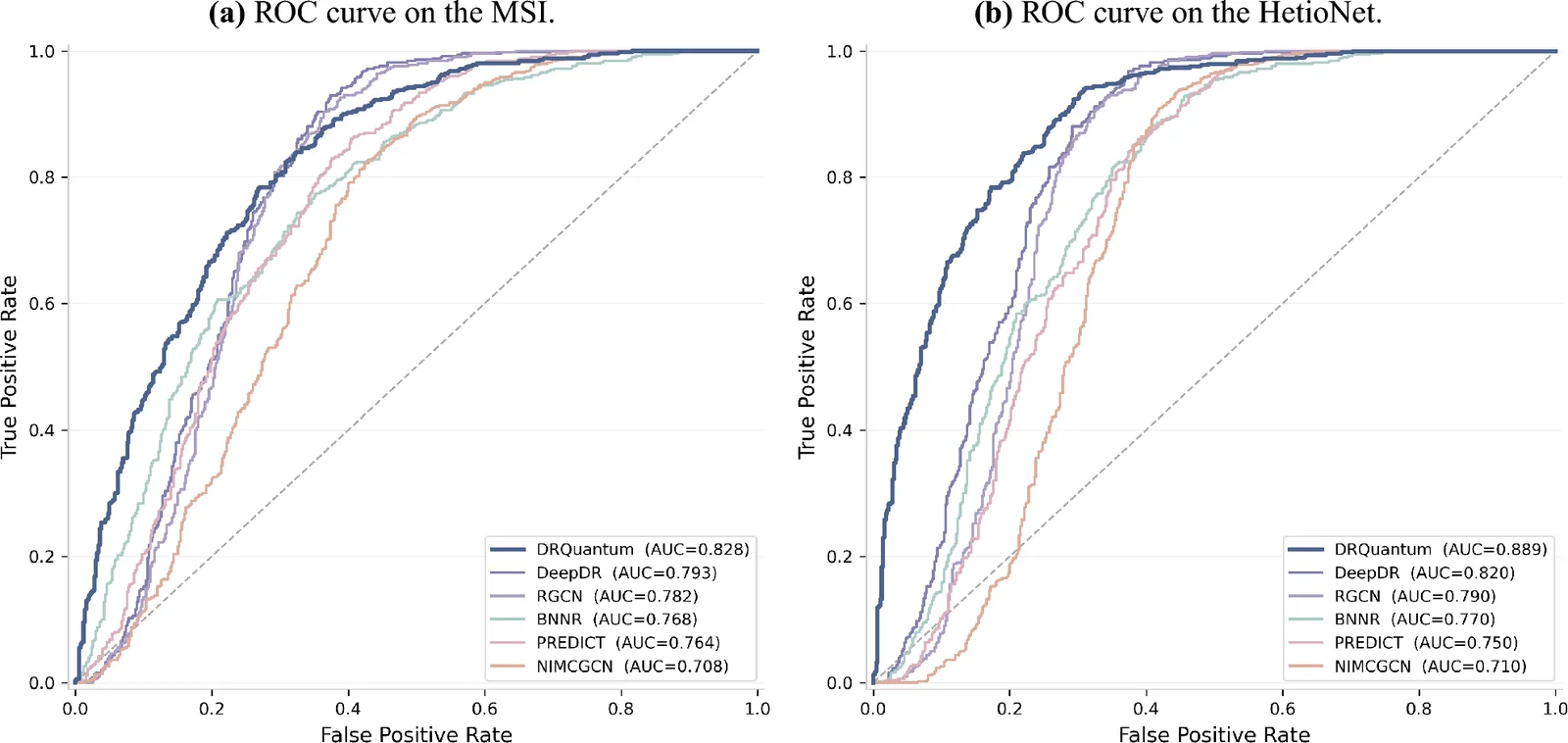
Comparison of ROC curves for each model on the two biomedKGs.

**Fig. 4:**
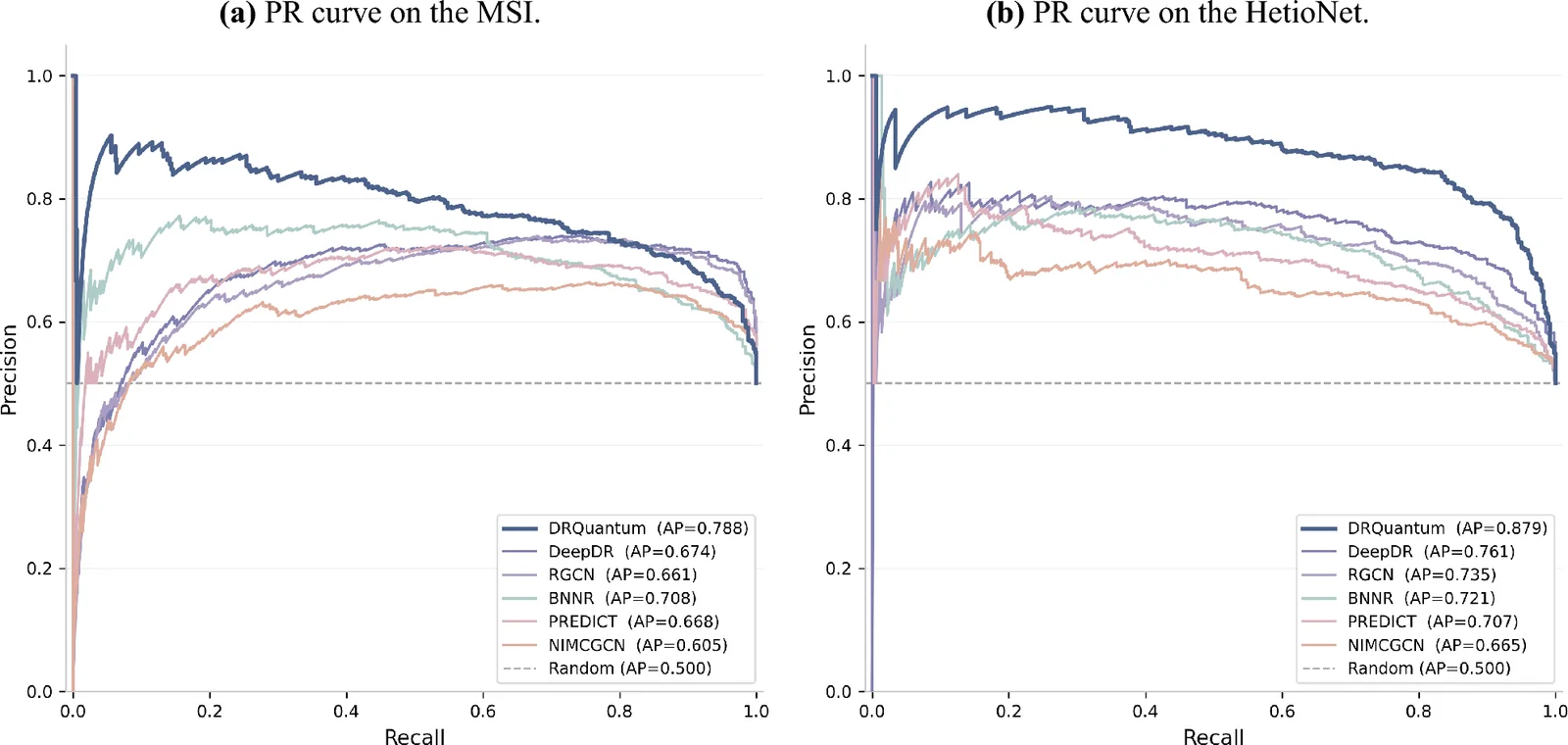
Comparison of PR curves for each model on the two biomedKGs.

**Fig. 5:**
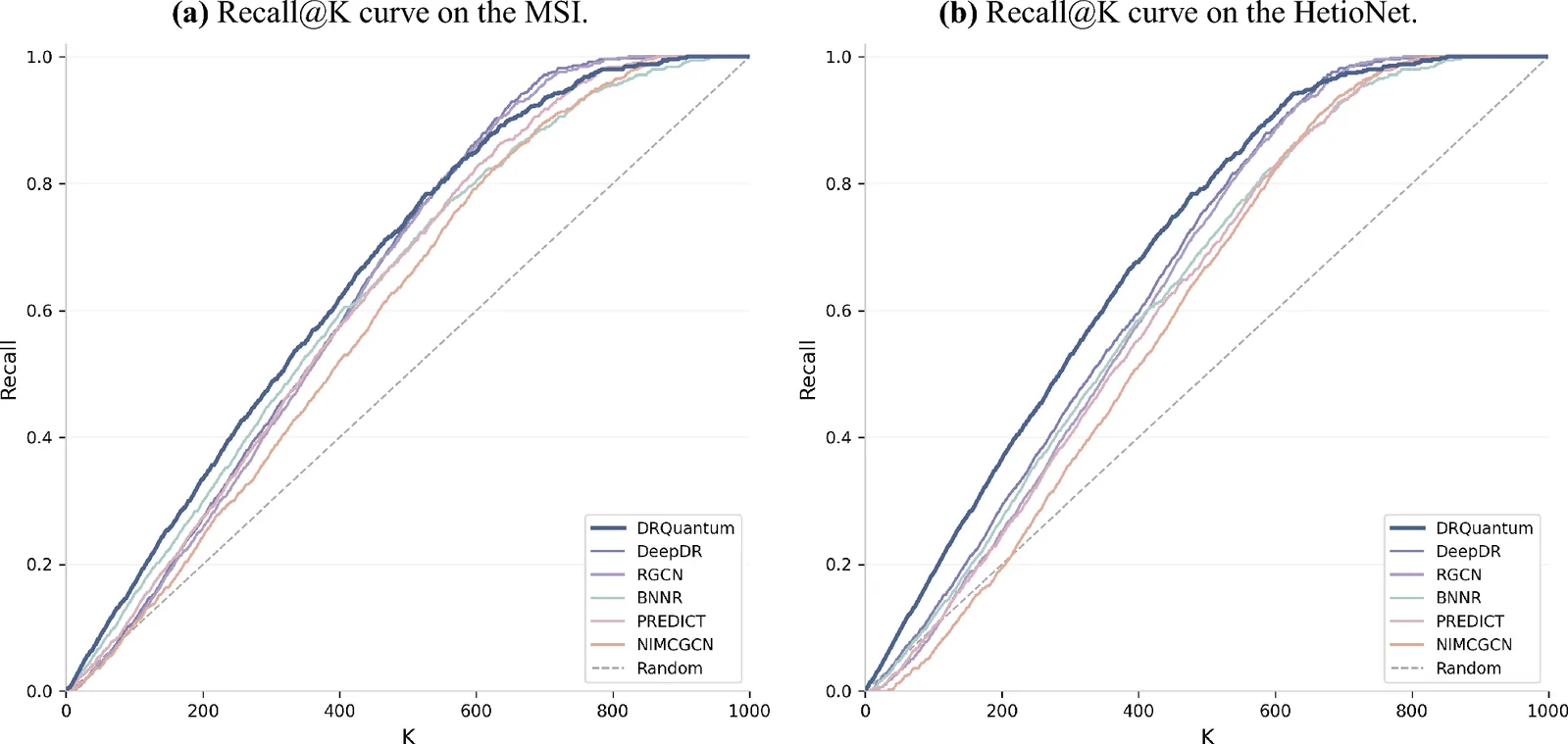
Comparison of Recall@K curves for each model on the two biomedKGs.

Overall, these results demonstrate that DRQuantum consistently outperforms all competing methods across both datasets and evaluation metrics. The advantage is particularly pronounced in AUPRC, indicating that DRQuantum is especially effective in prioritizing positive drug–disease associations under sparse and highly imbalanced settings. The consistent improvements across two independently constructed biomedical knowledge graphs further confirm the generalizability of the proposed framework. The ROC, PR, and Recall@K curves further corroborate these findings (Figs. 3, 4 and 5).

### Ablation studies

To further evaluate the contribution and necessity of each core component in DRQuantum, ablation studies were conducted from two perspectives. In the embedding module ablation, the downstream XGBoost classifier was fixed while the RED embedding algorithm^35^ (see Methods section) was replaced with several representative graph embedding methods for comparison. In the classifier module ablation, the RED embedding algorithm was fixed while XGBoost was replaced with various mainstream machine learning classifiers. Both experiments were conducted on the MSI and HetioNet datasets and evaluated using AUROC, AUPRC, and Accuracy, to validate the predictive performance and generalizability of DRQuantum’s architectural design across diverse network structures.

#### Ablation studies on the embedding module

**Fig. 6:**
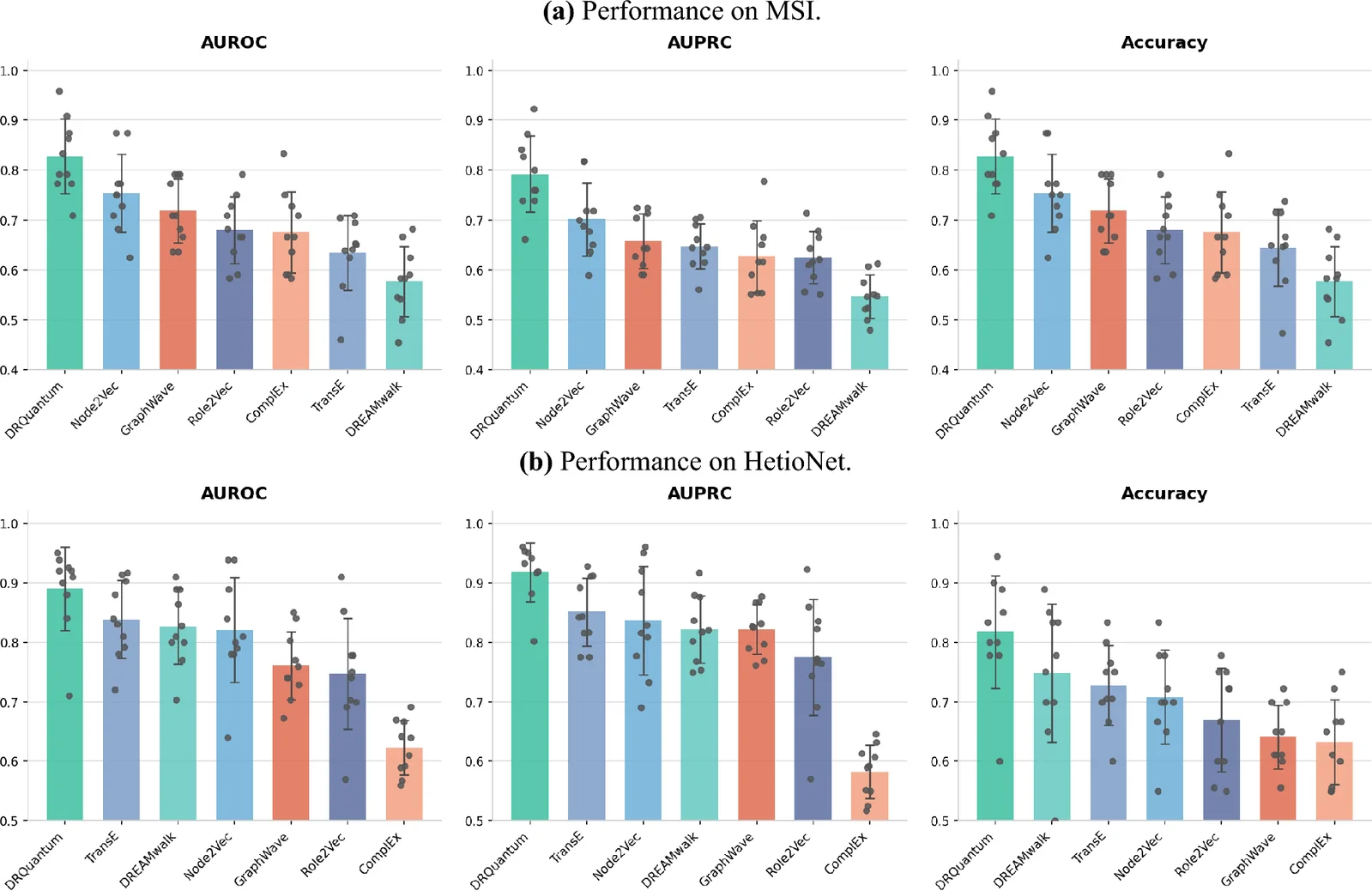
Effects of different node embedding methods on DDA prediction performance. Bar height represents the mean value derived through *n=10* independent experiments, and error bars denote the standard deviation across 10-fold cross-validation experiments. Individual dots represent results from each repetition.

The compared methods included random walk-based algorithms, such as Node2Vec^11^, Role2Vec^12^, and DREAMwalk^13^, which learn node representations through different random walking strategies. Knowledge graph embedding models, including TransE^14^ and ComplEx^14^, were also evaluated for their capability to capture complex relational patterns in low-dimensional embedding spaces. In addition, GraphWave^15^ was incorporated as a structural embedding approach that characterizes node roles and structural similarities through spectral graph wavelets. Experimental results on both the MSI and Hetionet datasets indicate that the proposed DRQuantum embedding consistently achieves superior predictive performance across diverse network structures (Fig. 6).

Classical random walk methods (Node2Vec, Role2Vec, and DREAMwalk) show relatively limited performance on both datasets, with DREAMwalk yielding an AUROC of 0.577 and AUPRC of 0.547 on MSI, and an AUROC of 0.826 and Accuracy of 0.748 on HetioNet, suggesting that classical Markovian processes, constrained by localized diffusion and independent path sampling, have limited capacity to retain long-range pathways across multi-layered sparse topologies. GraphWave similarly shows constrained performance on both benchmarks, achieving an AUROC of 0.719 on MSI and 0.761 on HetioNet, with AUPRC of only 0.658 and 0.821 respectively, suggesting that purely local structural information remains insufficient for heterogeneous drug repurposing tasks. Knowledge graph embedding methods (TransE and ComplEx) face notable challenges on both datasets, with ComplEx achieving an AUROC of only 0.675 on MSI and 0.623 on HetioNet, and TransE reaching an AUROC of 0.635 on MSI and 0.838 on HetioNet, as their rigid translational or bilinear constraints present difficulties in modeling the complex, non-linear associations characteristic of biological networks. In contrast, DRQuantum leverages quantum superposition to simultaneously explore parallel trajectories, enabling more effective integration of high-order topologies and semantic heterogeneities across diverse network structures.

#### Ablation studies on the classifier module

**Fig. 7:**
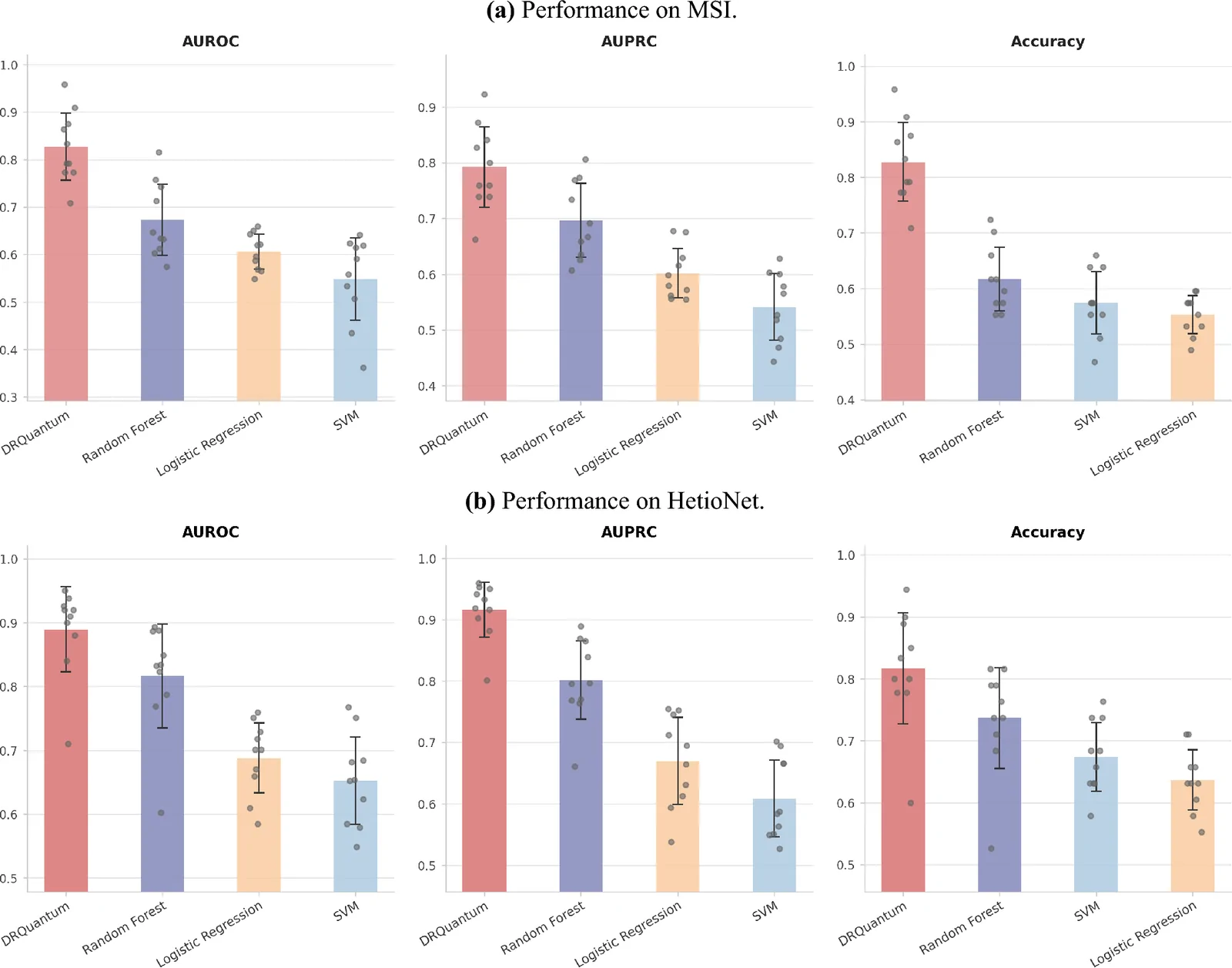
Effects of different downstream classifiers on DDA prediction performance. Bar height represents the mean value derived through *n=10* independent experiments, and error bars denote the standard deviation across 10-fold cross-validation experiments. Individual dots represent results from each repetition.

In the classifier module ablation, the RED embedding was kept fixed while XGBoost was replaced with Random Forest^36^, Support Vector Machine (SVM)^37^, and Logistic Regression (LR), representing ensemble, margin-based, and linear classification paradigms, respectively. As shown in Fig. 7, XGBoost consistently achieves the best performance across both datasets, highlighting its stronger capability to capture complex nonlinear relationships from quantum-walk embeddings.

Linear and kernel-based models (LR and SVM) show notable performance limitations, with SVM achieving an AUROC of only 0.549 on MSI and an Accuracy of 0.674 on HetioNet, suggesting that shallow decision boundaries are insufficient for modeling the non-linear geometric dependencies captured by quantum-walk trajectories. Random Forest demonstrates greater resilience on the denser HetioNet network with an AUROC of 0.816, but its performance drops to 0.673 on the sparser MSI network, indicating that standard bagging operations lack the adaptive capacity to handle varying network densities consistently. These results confirm that DRQuantum’s classification performance relies not only on high-quality quantum-walk embeddings, but also on the synergistic integration of an optimized downstream classifier.

### Parameter sensitivity analysis

In this section, we conduct sensitivity analyses on two key hyperparameters of DRQuantum: the global walk step $d_g$ and the similarity threshold, which controls the sparsification of the molecular similarity network. Since the diameter of the MSI network is 8, we set *d=8* to ensure that the quantum walk traverses the full topological extent of the network, and fix this value throughout all experiments while varying each hyperparameter over a representative range.

**Fig. 8:**
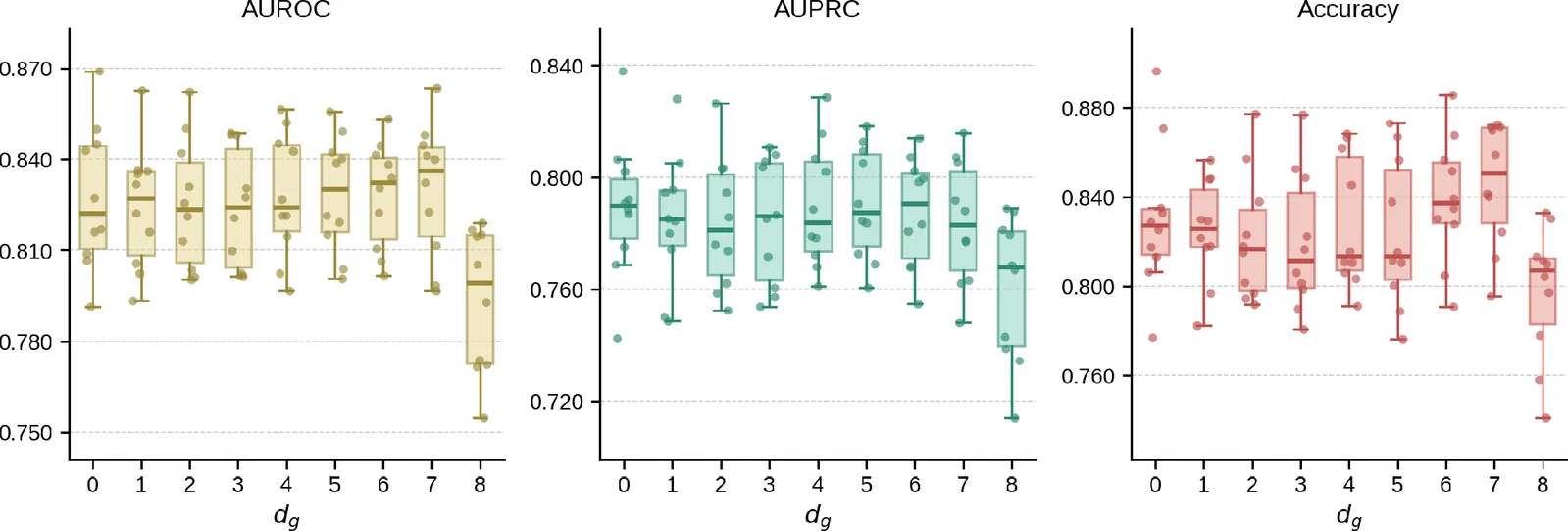
Parameter sensitivity analysis of DRQuantum to $d_g$. DDA prediction performances (Accuracy, AUROC, and AUPRC) following the change in $d_g$. Global quantum walk step $d_g$ was fixed at 4. All data have been derived through *n = 10* independent experiments, individual dots represent results from each repetition.

We vary $d_g \in \{0, 1, 2, 3, 4, 5, 6, 7, 8\}$ with $d_l = d - d_g$, focusing on $d_g$, which governs the balance between global and local structural information in the node embedding. As shown in Fig. 8, all three evaluation metrics remain stable across $d_g = 0$ to $d_g = 7$, demonstrating that DRQuantum is robust to this parameter over a wide range. Performance degrades notably at $d_g = 8$ (i.e., $d_l = 0$), confirming that local structural information captured by the biased quantum walk is indispensable. We therefore set $d_g = d/2 = 4$ as the default, which allocates equal dimensions to global and local components and achieves a principled balance between macro-level topology and micro-level structural context.

**Fig. 9:**
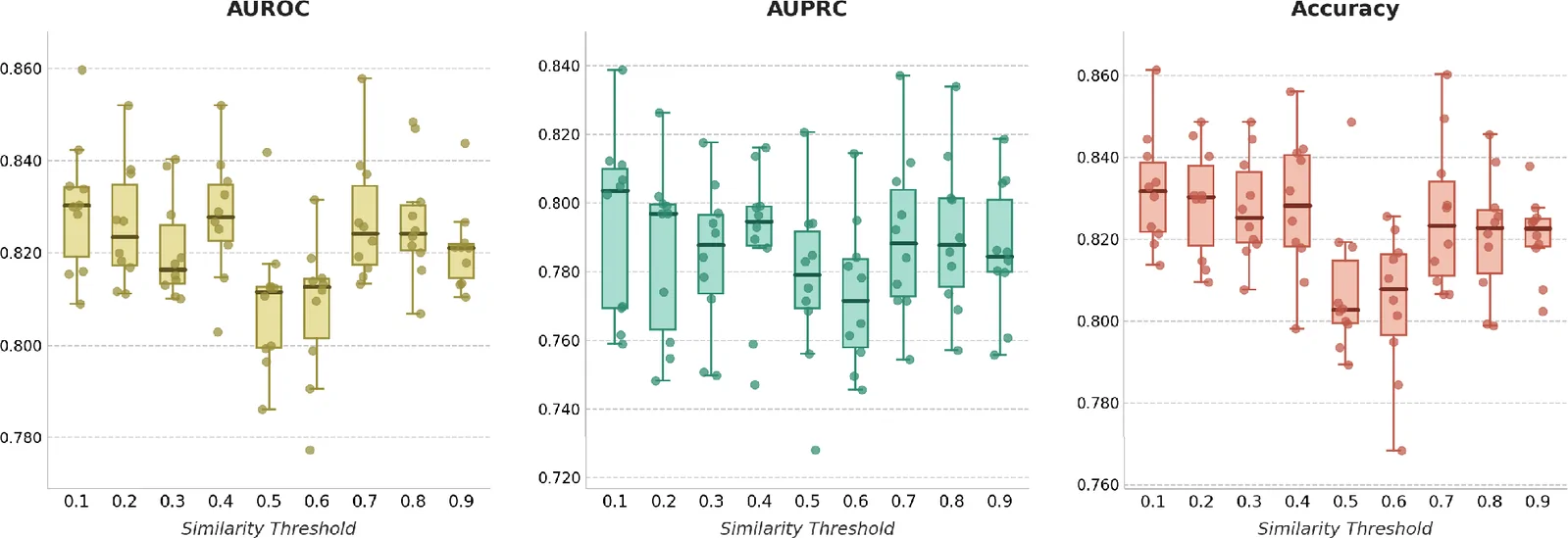
Parameter sensitivity analysis of DRQuantum to similarity threshold. DDA prediction performances (Accuracy, AUROC, and AUPRC) following the change in similarity threshold. All data have been derived through *n=10* independent cross-validation experiments; individual dots represent results from each fold.

The similarity threshold governs the connectivity of the constructed graph: a higher threshold retains fewer edges, resulting in a sparser graph with diminished structural information propagation between nodes. An excessively low threshold introduces redundant edges that compromise the independence of data partitioning, whereas an excessively high threshold leads to near-disconnected graphs and substantial loss of structural information. Intermediate thresholds offer a favorable trade-off between these two extremes. As shown in Fig. 9, across all three evaluation metrics, model performance does not degrade monotonically with increasing threshold but instead exhibits a non-monotonic pattern, with consistently higher and more stable performance at lower thresholds (0.1–0.4), a notable dip in the mid-range (0.5–0.6), and partial recovery at higher values albeit with greater inter-fold variability. Taking both predictive performance and cross-fold stability into account, we adopt a similarity threshold of 0.4 as the default configuration, as this value maintains sufficient graph connectivity to support effective structural feature propagation while preserving the integrity of data partitioning.

### Complexity analysis

As for the complexity of DRQuantum, DRQuantum algorithm totally walks for $O(D\cdot \vert V \vert + d_g)$ steps of DTQW, and its complexity mainly depends on the cost of DTQW. Each step of DTQW is a unitary transformation. In quantum computation, conducting such a unitary transformation is *O*(1) so one step of DTQW is *O*(1). Consequently, the theoretical complexity of DRQuantum is $O(D\cdot \vert V\vert )$ in quantum computation, almost linear to the number of nodes. However, similar to other quantum methods, current technologies compel us to simulate DTQW on classical computers. According to the previous study, simulating one step of DTQW is $O(\vert V\vert ^4 )$^38^, so simulating DRQuantum on classical computers grows to $O(D\cdot \vert V \vert ^ 5)$. For some methods of role embedding, including Node2vec and GraphWave, the complexity is linear to the number of edges $O(\vert E\vert )$; for other methods like Role2vec, the time complexity may be sacrificed for reducing its space cost. Compared with those methods, the simulation complexity of DRQuantum may be high, but the theoretical complexity of DRQuantum is only linear to the node number $O(\vert V \vert )$, which is also very competitive. Note that it is important to distinguish the real complexity in quantum computation from the simulation. The original theoretical complexity of DRQuantum in quantum computation is only $O(D\cdot \vert V\vert )$, and its high complexity of simulation is temporary. Despite the simulation, DRQuantum can be vastly optimized if directly conducted rather than simulated, which can be soon realized by future technologies.

### Algorithm visualization

**Fig. 10:**
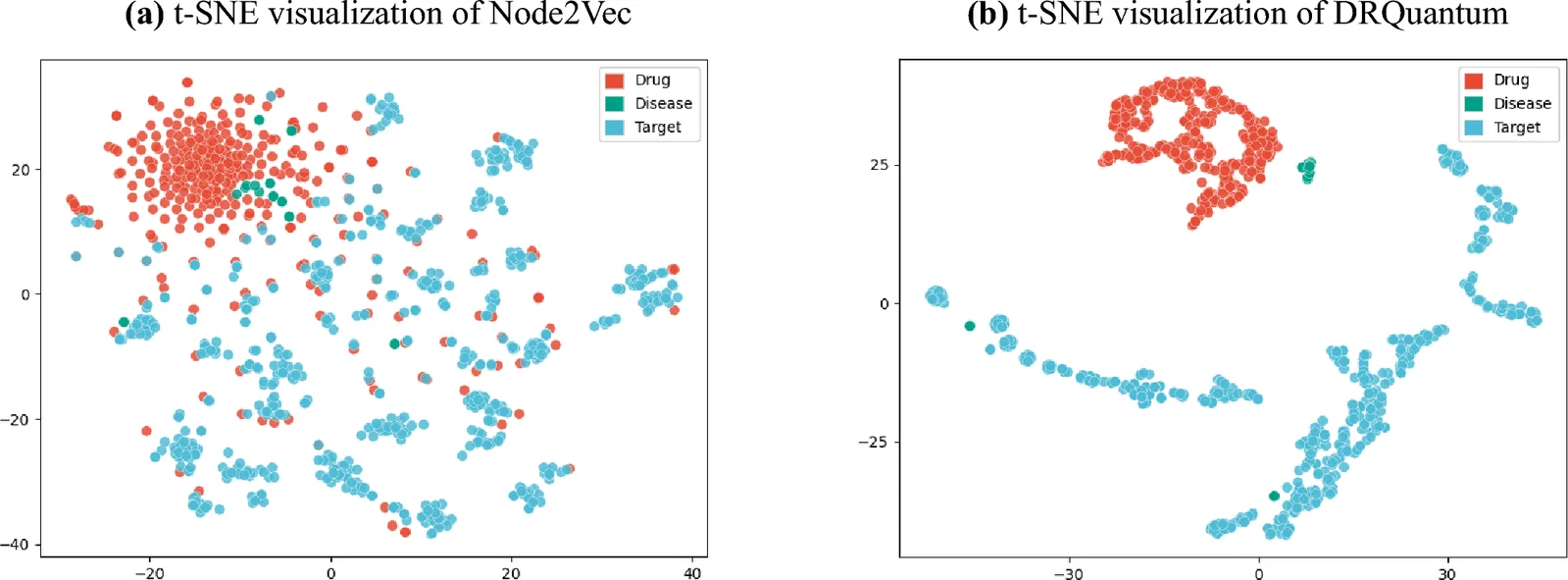
Random walk based embedding algorithm versus quantum walk based embedding algorithm. t-SNE visualization of node embeddings generated by Node2Vec (**a**) and DRQuantum (**b**) on the MSI network. Quantum walk based embeddings achieve clearer separation among drug, target, and disease nodes than their random walk based counterparts.

In Fig. 10, we present the t-SNE visualization results of two different embedding algorithms, Node2Vec and DRQuantum.

Figure 10a shows the embedding results of the Node2Vec algorithm based on random walks. It is evident from the figure that there is significant overlap among diseases, drugs, and targets, particularly between drugs and targets. This overlap indicates that the Node2Vec algorithm has limitations in distinguishing between different categories, failing to effectively separate different types of data points. This may be due to the limitations of the random walk method in capturing the complex relationships between nodes.

Figure 10b shows the embedding results of the DRQuantum algorithm based on quantum walks. Compared to the Node2Vec algorithm, the DRQuantum algorithm can more clearly separate different categories of data points. Specifically, the separation between drugs and targets is more pronounced, and the distribution of targets is more dispersed. This indicates that the DRQuantum algorithm has stronger feature extraction and classification capabilities when dealing with complex network structures.

The parallelism of quantum walks allows them to traverse multiple paths simultaneously. This characteristic enables quantum walks to capture the global structural information of the network more effectively, thereby enhancing the quality of the embedded representations. This is fundamentally different from random walks, which can only move along a single path at each step and thus show certain limitations in handling complex relationships.

### Case studies

To further corroborate the predictive capability of the DRQuantum algorithm, we conducted in-depth case studies on hypertension and diabetes, both of which necessitate prolonged management and symptomatic therapy. Our primary aim is to propose the redirection of existing medications, and thus, we identified potential drug candidates for these diseases within the MSI network. For each condition, we employed the DRQuantum training method to compute drug-disease association probabilities for all unlabeled drugs, repeating this process ten times using varying negative sets. Following the calculation of the average probabilities, we narrowed down our search to the top 15 drugs with the highest probabilities as prime candidates for drug repurposing. Tables 1 and 2 present the average probabilities for each drug, alongside their original intended uses and supporting literature that validates the repurposing evidence.

$\textbf{Hypertension}$ In the hypertension drug repurposing predictions, our focus was on the top 15 candidate drugs. Each drug is accompanied by its standard name, predicted score, and supporting evidence from literature. Aranidipine, a drug traditionally used for hypertension, emerged with the highest predicted score, suggesting exceptional potential efficacy. Cyproheptadine, despite its original indications for anorexia and allergic symptoms, garnered a predicted score of 99.3% for hypertension treatment. Prior research suggests that Cyproheptadine may influence blood pressure via its side effects on vascular regulation. Similarly, Nintedanib, which is primarily used for idiopathic pulmonary fibrosis and non-small cell lung cancer, showed a high predicted probability of 99.3% for hypertension treatment, likely attributable to its antifibrotic and anti-inflammatory properties. Macitentan and Sitaxentan, both originally intended for pulmonary arterial hypertension, achieved high scores of 98.9%, indicating their potential effectiveness in hypertension management. Notably, Aranidipine, a well-known hypertension treatment, has now been predicted to have a 99.8% average probability of effectiveness in hypertension management, further validating its efficacy. Cyproheptadine, primarily used for anorexia and allergic symptoms, has demonstrated a 99.3% probability of being effective for hypertension in this study, potentially broadening its clinical applications.

**Table 1:** Top 15 drug repurposing candidates identified by DRQuantum for Hypertension.

Rank	Drug	Original indication	Avg. prob. (%)	Evidence
1	Aranidipine	Essential Hypertension	99.8	^39^
2	Cyproheptadine	Anorexia, Allergic Symptoms	99.3	^40^
3	Nintedanib	Idiopathic Pulmonary Fibrosis, Non-Small Cell Lung Cancer	99.3	^41^
4	Macitentan	Pulmonary Arterial Hypertension	98.9	^42^
5	Sitaxentan	Pulmonary Arterial Hypertension	98.9	^43^
6	ACT-132577	Pulmonary Arterial Hypertension	98.9	^44^
7	Imidapril	Essential Hypertension, Congestive Heart Failure	98.8	^45^
8	Quinaprilat	Essential Hypertension, Congestive Heart Failure	98.8	^46^
9	Enalaprilat	Essential Hypertension, Congestive Heart Failure	98.8	^47^
10	Spirapril	Essential Hypertension	98.8	^48^
11	Remikiren	Hypertension, Congestive Heart Failure	98.7	^49^
12	Aliskiren-Hemifumarate	Essential Hypertension	98.7	^50^
13	Glycerin	Cerebral Edema	98.2	^51^
14	Quercetin	Cardiovascular Diseases	98.2	^52^
15	Insulin human	Diabetes	97.3	^53^

$\textbf{Diabetes}$ Among the outcomes of diabetes drug repurposing predictions, the top 15 candidate drugs exhibited remarkable potential. Macitentan, topping the list with a predicted score of 98.9%, originally intended for pulmonary arterial hypertension, may hold promise in diabetes treatment due to its role in vascular function regulation. Ustekinumab, originally used for psoriasis and Crohn’s disease, also scored highly at 98.7% for diabetes treatment, likely due to its immune modulation effects suggested by the literature. Moreover, Chlorpropamide, Nateglinide, and Glimepiride, all current diabetes treatment, garnered high probabilities, further confirming their efficacy. This suggests that many existing diabetes therapies could yield enhanced outcomes through novel applications. Notably, 70% of the top 20 predicted drugs have been validated by clinical, preclinical, and other literature data. These drugs, with their unique roles in inflammation and immune regulation, could offer new treatment avenues and hope for diabetes patients, ultimately enhancing the diversity and precision of diabetes therapy.

**Table 2:** Top 15 drug repurposing candidates identified by DRQuantum for Diabetes.

Rank	Drug	Original indication	Avg. prob. (%)	Evidence
1	Macitentan	Pulmonary Arterial Hypertension	98.9	^54^
2	Ustekinumab	Psoriasis, Crohn’s Disease	98.7	^55^
3	Chlorpropamide	Diabetes Mellitus	98.6	^56^
4	Nateglinide	Diabetes Mellitus	98.6	^57^
5	Glimepiride	Diabetes Mellitus	98.6	^58^
6	Tolazamide	Diabetes Mellitus	98.6	^59^
7	Glipizide	Diabetes Mellitus	98.6	^60^
8	Acetohexamide	Diabetes Mellitus	98.6	^61^
9	Repaglinide	Diabetes Mellitus	98.6	^62^
10	L-Cysteine	Antioxidant Support, COPD, Acetaminophen Toxicity	98.5	^63^
11	Telithromycin	Pneumonia	98.0	–
12	Glutathione	Alzheimer’s Disease, Parkinson’s Disease	98.0	^64^
13	Chondroitin Sodium	Osteoarthritis	97.8	^65^
14	Rilonacept	Cryopyrin-Associated Periodic Syndromes	97.2	^66^
15	Dehydroepiandrosterone	Allergic Rhinitis, Urticaria	96.5	^67^

**Fig. 11:**
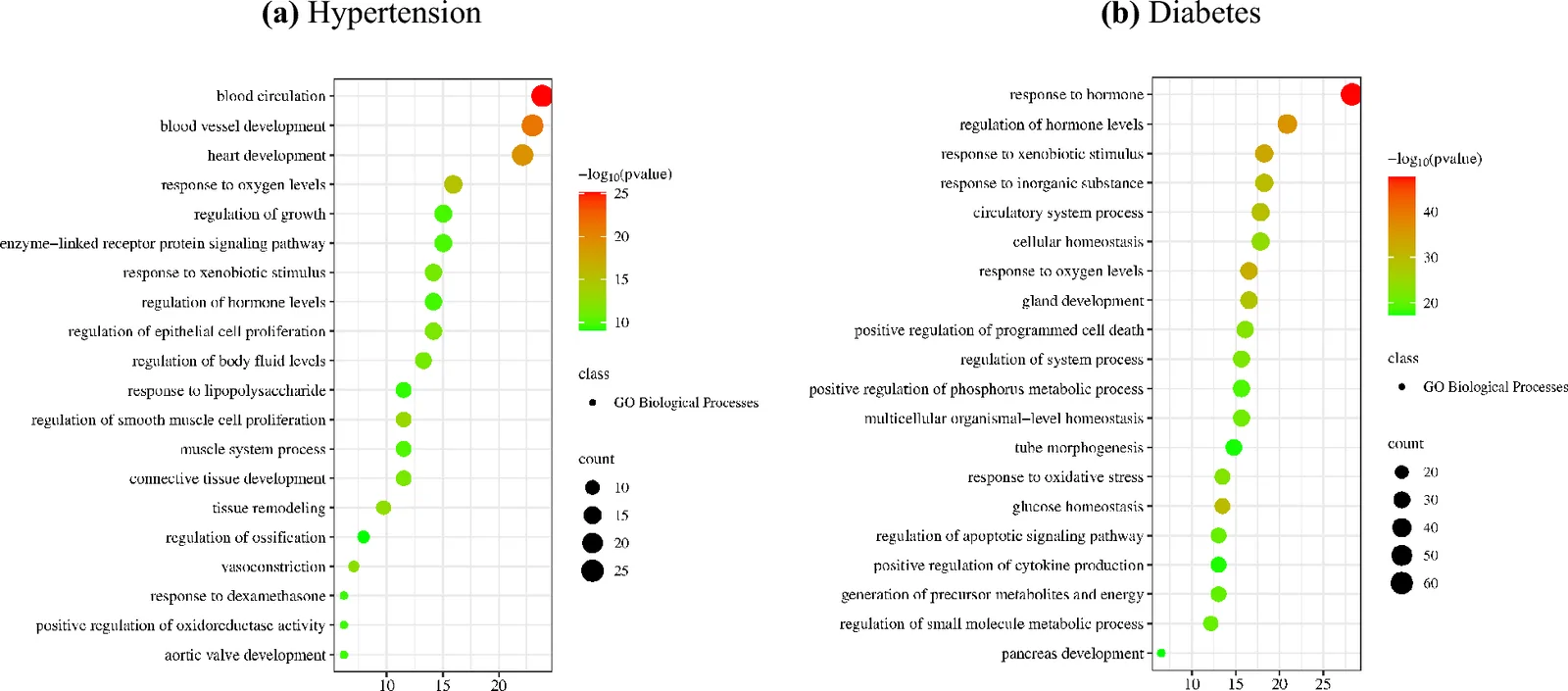
Gene Ontology (GO) enrichment analysis of target genes associated with the top 15 drug repurposing candidates predicted by DRQuantum for hypertension and diabetes. Bubble size represents the number of enriched genes, and the color gradient indicates the statistical significance level ($-\log _{10}p$-value).

To further investigate the biological relevance of the drug-disease associations predicted by DRQuantum, we performed Gene Ontology (GO) enrichment analysis on the candidate target genes associated with hypertension and diabetes (Fig. 11). Specifically, the top 15 candidate drugs predicted by DRQuantum for each disease were first identified, and their biological targets were subsequently retrieved from the DrugBank database. The union of these target genes was then submitted to GO enrichment analysis (*p<0.05*). The results demonstrate that these genes are significantly enriched in several critical biological processes (BP). Specifically, for hypertension, the genes are predominantly involved in “response to hormone” and “response to endogenous stimulus,” which are essential for the physiological regulation of vascular tone. For diabetes, the enrichment terms are primarily concentrated in “positive regulation of cytokine production” and “inflammatory response,” reflecting the pivotal role of inflammation in insulin resistance and metabolic dysfunction. Furthermore, Molecular Function (MF) analysis reveals that these genes are extensively involved in receptor binding and signal transduction activities. These enrichment findings suggest that the drugs identified by DRQuantum are not only statistically significant but also biologically aligned with the pathophysiological mechanisms of the target diseases.

## Discussion

The superior performance of DRQuantum underscores the potential of quantum walks to capture complex topological features in multi-layered heterogeneous biological networks, particularly through the exploitation of quantum superposition and coherence for global feature extraction. Despite these promising results, several critical challenges and future research directions warrant further investigation. First, the computational complexity of simulating discrete-time quantum walks (DTQW) on classical architectures remains a significant bottleneck ($O(D \cdot |V|^5)$), which may limit its scalability to ultra-large-scale omics datasets involving millions of nodes. Future efforts should focus on developing more efficient quantum operator approximation algorithms or exploring deployment on emerging quantum hardware to realize the theoretical advantage of linear complexity. Second, while the current framework integrates structured similarity data, incorporating dynamic multi-omics information—such as single-cell transcriptomic profiles or metabolic flux data—could significantly enhance the model’s sensitivity in predicting individualized drug responses. Furthermore, improving the biological interpretability of quantum embeddings remains a key challenge; establishing direct mappings between quantum state sequences and specific molecular mechanisms would provide deeper insights into drug action. Finally, extending DRQuantum to address the complexities of synergistic drug combination prediction and the assessment of off-target toxicities in repurposing candidates will be essential for its translation into clinical precision medicine.

## Conclusions

This study presents a novel method for drug repurposing— DRQuantum, marking the first application of quantum algorithms in the field of drug repositioning. Unlike traditional random walks, DRQuantum leverages quantum entanglement to simultaneously explore multiple paths within a network, thereby accelerating the traversal of large-scale heterogeneous biological networks. This innovative approach addresses the significant challenge of accurately capturing the complex structures inherent in these networks.

Compared to classical algorithms, our algorithm integrates both global and local information for node embedding. By constructing a multi-layer heterogeneous network that encompasses drug-drug, disease-disease, and protein-protein interactions, and applying quantum walks to generate low-dimensional feature representations of nodes, DRQuantum captures both the overall structure and local details of the network. This dual consideration of global and local network structures enhances the accuracy of drug-disease relationship predictions, providing a robust framework for effective drug repurposing. Comparative analyses with other state-of-the-art (SOTA) algorithms, including similarity- and matrix-based methods, random walk-based methods, knowledge graph embedding-based methods, deep learning and graph neural network (GNN)-based methods, demonstrate the superior performance of DRQuantum. Evaluation results on the MSI and Hetionet databases indicate that DRQuantum consistently outperforms these SOTA algorithms across key metrics such as AUROC, AUPRC, and accuracy.

The findings of this research highlight the significant potential of quantum computing techniques in the field of computational drug repurposing. By harnessing the unique advantages of quantum walks, DRQuantum not only enhances the efficiency and accuracy of drug repurposing predictions but also paves the way for future advancements in this area.

## Methods

### Drug and disease similarity

We calculated drug/disease similarity based on the ATC coding system and MeSH coding system encodings to construct a drug/disease interaction network. Various measures have been proposed to assess the similarity of entities in biomedical ontologies. Some of these measures rely on comparing the information content (IC) of the entities. IC provides a metric of how informative an entity c is, based on its occurrence frequency in a given biomedical corpus. The more frequently an entity appears, the less informative it is. Hence, a smaller IC value is assigned to the entity. Calculating the IC value of an entity directly from a tree-structured hierarchy, rather than a given corpus, can be achieved by counting the number of child nodes Nchild. The IC value of a term in a hierarchy structure can be defined as

$$\begin{aligned} \textrm{IC} (c)=1-\frac{log(N_{child}(c)+1)}{log(N_{child}(root))}, \end{aligned}$$

where $N_{child}(c)$ is the number of child nodes of term *c* and $N_{child}(root)$ is the total number of terms in the hierarchy.

According to previous research, given the IC value of two entities $c_1$, $c_2$ and their most informative common ancestor (MICA), the distance between the two entities can be defined as

$$\begin{aligned} d(c_1,c_2)=\textrm{IC}(c_1)+\textrm{IC}(c_2)-2\textrm{IC}(\textrm{MICA}(c_1,c_2)). \end{aligned}$$

Since $\max (\textrm{IC}) = 1$, the maximum value of the semantic distance between two entities is 2. To transform the distance measure into similarity value in the range of [0,1), the similarity measure can be defined as below:

$$\begin{aligned} \textrm{sim}(c_1,c_2)=1-\frac{d(c_1,c_2)}{2}. \end{aligned}$$

The similarity measure is calculated pairwise for all drugs and diseases of the three biomedical knowledge graphs (biomedKGs), based on their respective drug/disease hierarchies. A user-defined cutoff may be introduced to eliminate pairs with a similarity below a given cutoff value, in order to reduce noise and improve computational efficiency. For our study, the cutoff is set to 0.4 for all biomedKGs, and all similarities below this threshold are masked. In other words, drug/disease pairs with a similarity of less than 0.4 will not be connected in the biomedKGs, while those with a similarity greater than 0.4 will be linked with edges.

### Quantum walks

Discrete-time quantum walks (DTQW), initially proposed by Y. Aharonov et al. in 1993^23^, can be viewed as the quantum analogue of classical random walk. Serving as a fundamental model in quantum computing, DTQW finds diverse applications in quantum algorithms, encompassing Grover’s search algorithms^24^, quantum classification algorithms, and quantum clustering algorithms. Depending on the type of evolution, DTQW can be categorized into coined quantum walks^25^ and Szegedy quantum walks^26^. In this paper, our focus is solely on Szegedy quantum walks. In contrast to classical random walks, the walker in quantum walks exists in a superposition state, traversing all potential paths simultaneously. This distinctive trait ensures that quantum walks retain path information, thereby offering a quadratic speedup effect.

For a graph *G = (V,E)* with the number of nodes $\vert V\vert =N$, the Szegedy quantum walks on the graph *G* is defined on the Hilbert space $\mathcal {H}=\mathcal { H}_P \otimes \mathcal {H}_P$, which is the tensor of two position spaces $\mathcal {H}_P$ spanned by the nodes $\{\vert v \rangle : v=1,\cdots ,N\}$. The element $\vert i,j\rangle$ in $\mathcal {H}$ represents that the walker is currently at node *i* and the next step will be towards node *j*. The state of the walker at time *t* can be expressed as a superposition of $\left\{ \vert i,j\rangle :i,j\in V \right\}$, i.e. $\vert \Psi (t)\rangle =\sum _{i,j=1}^{N} \Psi _{ij}(t)\vert i,j\rangle$, where the coefficients $\Psi _{ij}(t)$ satisfy the normalization condition $\sum _{i,j=1}^{N} \vert \Psi _{ij}(t) \vert ^2=1$ for each *t*. According to the principles of quantum mechanics, the probability of the walker being at node *v* at time *t* is $p_v^t=\sum _{j=1}^{N}\vert \Psi _{ij}(t) \vert ^{2}$.

Quantum walk contains two crucial components: the initial state and the unitary evolution operators. Typically, the initial state of a quantum walk $\vert \Psi (0)\rangle$ is specified based on the problem at hand. The evolution of Szegedy quantum walk is characterized by two unitary operators: the Grover operator and the swap operator. The Grover operator, widely utilized in Grover’s search algorithms, is defined as:

$$\begin{aligned} G=2\sum _{v=1}^{N} \vert \psi _v\rangle \langle \psi _v\vert -I, \end{aligned}$$

where the projector states $\vert \psi _v\rangle$ are defined as $\vert \psi _v\rangle =\sum _{i,j=1}^{N}a_{ij}\vert i,j\rangle$ and

$$\begin{aligned} a_{ij}=\left\{ \begin{array}{ll} \frac{1}{\sqrt{d(v)}},& i=v \ \text {and}\ (i,j)\in E \\ 0,& \text {otherwise}. \end{array}\right. \end{aligned}$$

where *d*(*v*) represents the degree of vertex *v*. Then, the swap operator, *S*, is used to swap the states of two nodes:

$$\begin{aligned} S=\sum _{i=1}^{N} \sum _{j=1}^{N}\vert j,i\rangle \langle i,j\vert . \end{aligned}$$

By combining the two former operators, we obtain the unitary operator $U_W=SG$. The evolution of the state can then be described as:

$$\begin{aligned} \vert \Psi (t+1)\rangle =U_W\vert \Psi (t)\rangle =(U_W)^t\vert \Psi (0)\rangle . \end{aligned}$$

Specifically, when the unitary operator $U_W$ is applied to a basis state, it evolves the state according to the defined unitary operation.

$$\begin{aligned} U_W\vert i,j\rangle =\left( \frac{2}{d(j)} -1 \right) \vert j,i\rangle +\frac{2}{d(j)}\sum _{k\ne i,(j,k)\in E} \vert j,k\rangle . \end{aligned}$$

### Quantum network embedding algorithms

In essence, leveraging the correlation that nodes with similar roles exhibit analogous evolutions and quantum walk behaviors, we can represent the network information of nodes through quantum walks on the graph *G*, thereby achieving node embedding. By implementing quantum walks within the network, we can manipulate the probability distribution of the initial state at each node to extract latent semantic information from these nodes. Wang et al.^35^ introduced the RED algorithm, a method that harnesses the properties of discrete-time quantum walks to learn role embedding from both global and local perspectives. The primary objectives of node embedding encompass both global role information representation and local role information representation. The former aims to analyze roles from the vantage point of the entire network, while the latter focuses on local structures, highlighting the correlations between the target node and its immediate neighbors.

$\mathbf {Global\ role\ information\ representation}$ The objective of global role information is to dissect node data from the perspective of traversing the entire graph. Unlike the traditional random walk, the quantum walk is a more suitable approach for capturing global insights. Classical random walks, inherently, are confined to traversing a single path at a given time, thus rendering them inadequate for a comprehensive examination of the entire network. Conversely, under the premises of quantum mechanics, a walker can exist in a state of superposition, enabling the involvement of all nodes into global evolution. This allows quantum walks to traverse all potential paths simultaneously, without forfeiting any path information. Initially, all nodes engage in the evolutionary process. During each iteration, the walker concurrently alters its position and direction across all nodes. Consequently, these evolving attributes mirror the interactions between nodes and depict the relationship between individual nodes and the entire graph. This interconnection unveils the node’s standing within the context of the entire graph, thereby allowing us to leverage evolving features to represent global role information. Given the wealth of information encoded in quantum walks, a mere few steps within the period suffice. Therefore, we initially conduct a quantum walk on the unbiased state $\vert \Phi (0)\rangle$ for $d_g$ steps (in this paper we set $d_g = 4$), where

$$\begin{aligned} & \vert \Phi (0)\rangle =\sum _{i,j=1}^{N} \Phi _{ij}(0)\vert i,j\rangle , \\ & \Phi _{ij}(0)=\left\{ \begin{array}{ll} \frac{1}{\sqrt{N\cdot d(i)} }, & (i,j)\in E\\ 0,& \textrm{otherwise} \end{array}\right. \end{aligned}$$

this unbiased probability setting allows for a more thorough exploration of global information. We then utilized $p_v^t=\sum _{j=1}^{N} \vert \Phi _{vj}(t) \vert ^2$ to compute the probability $p_v^t$ for each node *v* at each step *t*. Subsequently, we generated the probability sequence of *v* over $d_g$ steps, serving as the global role information representation of the node, i.e.,

$$\begin{aligned} e_v^g=\left[ p_v^1,p_v^2,\dots ,p_v^{d_g} \right] . \end{aligned}$$

$\mathbf {Local\ role\ information\ representation}$ Local role information highlights local structures, emphasizing the correlations between a target node and its immediate neighbors. To accentuate the node *v* from a localized perspective, we elevated its probability, ensuring that *v* occupies a larger share in the walking evolution. Initially, it commences at node *v* with a probability of 1, and subsequently progresses to adjacent nodes with uniform probabilities. As it traverses all feasible paths concurrently, the walker reaches the first-order neighbors of *v* in the first step, second-order neighbors in the second step, and so on, ultimately encompassing all reachable nodes through successive steps. We terminated the walk after traversing all potential nodes by advancing for *D* steps, where *D* represents the graph diameter, i.e.,

$$\begin{aligned} D=\underset{(i,j)\in E}{\max }{\min } \left\{ d(i,j):\mathrm {path \ connect} \ i\ \textrm{and} \ j \right\} . \end{aligned}$$

We employed the *v*-biased state

$$\begin{aligned} & \vert \Psi _v(0)\rangle =\sum _{i,j=1}^{N} \Psi _{ij}(0)\vert i,j\rangle , \\ & \Psi _{ij}(0)=\left\{ \begin{array}{ll} \frac{1}{\sqrt{d(v)}},& i=v \ \textrm{and}\ (i,j)\in E\\ 0,& \mathrm {otherwise.} \end{array}\right. \end{aligned}$$

as the initial state for the quantum walk. During the biased quantum walk originating from node *v*, the accumulated probability offers insights into the proximity between the respective node and node *v*. Therefore, we defined the closeness between node *i* and node *v* as

$$\begin{aligned} c_v(i)=\sum _{t=1}^{D} \sum _{j=1}^{N} \vert \Psi _{ij}(t) \vert ^{2}, \end{aligned}$$

where $d_l$ represents the length of the walk (here we set it to 4). To compute the local role representation of *v*, we considered

$$\begin{aligned} e_v^l=\left[ \max _1 c_v,\max _2c_v,\dots ,\max _{d_l}c_v \right] , \end{aligned}$$

where $\max _k c_v$ retrieves the *k*-th largest closeness value among the neighbors of node *v*.

$\mathbf {RED \ quantum \ embedding\ algorithm}$ Given a graph *G=(V,E)*, for each node *v*, RED generates $e_v^g$ for global role information, and has obtained $e_v^l$ for local role information. Finally, RED combines $e_v^g$ and $e_v^l$ into a unified role embedding $e_v$ using a coupling function *f(· )*:

$$\begin{aligned} e_v=f(e_v^g,e_v^l). \end{aligned}$$

A simple yet efficient approach to fusing these embeddings is by concatenating $e_v^g$ and $e_v^l$, resulting in the length of $e_v$ is $d = d_g + d_l$. We set the parameters of RED to be *d* and $d_g$, and $d_l$ can be derived from *d* and $d_g$. For a fixed *d*, we can tailor the desired embedding by delicately balancing $e_v^g$ and $e_v^l$ through $d_g$ (where $0\le d_g\le d)$, with a higher $d_g$ resulting in a longer $e_v^g$ and a shorter $e_v^l$. In this paper, we set *d=8* and $d_g=4$.

### Experimental setup and cross-validation

Negative drug–disease pairs of equal number to the positive pairs were randomly sampled from unknown drug–disease pairs with no reported associations. A 10-fold cross-validation strategy was employed at the drug–disease pair level, repeated 10 times with different negative sets. All known drug–disease associations were strictly excluded from the training process during cross-validation to avoid information leakage.

## Data Availability

Each biomedKGs were retrieved from the corresponding GitHub repositories and through API calls; HetioNet (https://github.com/hetio/hetionet), MSI (https://github.com/snap-stanford/multiscale-interactome) and then further processed for this study.
